# Three Prizes

**Published:** 1859-10

**Authors:** 


*Three Prizes.*—Dr. Samuel Mallet, of Hartford, Ct., authorizes us to state that he will give *Twenty-five Dollars* to the student of the Graduating Class of 1859-60, of the Baltimore College of Dental Surgery, who shall present the best Thesis. Dr. A. A. Blandy also offers *Twenty-five Dollars* to the one who shall present the best specimen of artificial teeth, mounted upon the Cheoplastic principle, executed during the session; he also offers *Twenty-five Dollars* to the student who shall present the best specimen of continuous gum work.

These prizes will be awarded by a committee appointed by the gentlemen offering them, or in some other manner which shall be perfectly satisfactory to the candidates.

